# Dosimetric quantities and effective dose in medical imaging: a summary for medical doctors

**DOI:** 10.1186/s13244-021-01041-2

**Published:** 2021-07-13

**Authors:** Eliseo Vano, Guy Frija, Reinhard Loose, Graciano Paulo, Efstathios Efstathopoulos, Claudio Granata, Jonas Andersson, the European Society of Radiology (ESR)

**Affiliations:** 1 Radiology Department, Complutense University, 28040 Madrid, Spain; 2 Université de Paris, 12 Rue de l’École de Médecine, 75006 Paris, France; 3 Institute of Medical Physics, Hospital Nuremberg, Prof.-Ernst-Nathan-Str. 1, 90419 Nuremberg, Germany; 4 ESTESC-Coimbra Health School, Medical Imaging and Radiotherapy Department, Instituto Politécnico de Coimbra, Rua 5 de Outubro, S. Martinho Do Bispo, 3046-854 Coimbra, Portugal; 5 2Nd Department of Radiology, Medical Physics Unit, National and Kapodistrian University of Athens, Attikon University Hospital, 12462 Athens, Greece; 6 Department of Paediatric Radiology, Institute for Maternal and Child Health - IRCCS “Burlo Garofolo”, Trieste, Italy; 7 Department of Radiation Sciences, Umea University, Umeå, Sweden

**Keywords:** Dosimetric quantities, Diagnostic reference levels, Effective dose, Cumulative dose, Patient information

## Abstract

This review presents basic information on the dosimetric quantities used in medical imaging for reporting patient doses and establishing diagnostic reference levels. The proper use of the radiation protection quantity “effective dose” to compare doses delivered by different radiological procedures and different imaging modalities with its uncertainties and limitations, is summarised. The estimates of population doses required by the European Directive on Basic Safety Standards is commented on. Referrers and radiologists should be familiar with the dose quantities to inform patients about radiation risks and benefits. The application of effective dose on the cumulative doses from recurrent imaging procedures is also discussed.

** Patient summary**: Basic information on the measurement units (dosimetric quantities) used in medical imaging for reporting radiation doses should be understandable to patients. The Working Group on “Dosimetry for imaging in clinical practice” recommended that a brief explanation on the used dosimetric quantities and units included in the examination imaging report, should be available for patients. The use of the quantity “effective dose” to compare doses to which patients are exposed to from different radiological procedures and its uncertainties and limitations, should also be explained in plain language. This is also relevant for the dialog on to the cumulative doses from recurrent imaging procedures. The paper summarises these concepts, including the need to estimate the population doses required by the European Directive on Basic Safety Standards. Referrers and radiologists should be familiar with the dose quantities to inform patients about radiation risks and benefits.

## Key points

- Dosimetric quantities for reporting patient doses and comparing with diagnostic reference levels should be known by radiologists and radiographers.
- The radiation quantity “effective dose” with its uncertainties and limitations, may be used for comparison between different imaging modalities and procedures, and to explain the radiation risk to patients.
- The cumulative effective doses from recurrent imaging procedures in some groups of patients, may be considered in the justification and optimisation for the next imaging practices in these groups of patients.

## Introduction

This review summarises the basic information on the dosimetric quantities used in medical imaging and interventional procedures for reporting patient doses and comparing doses delivered by different radiological procedures and different modalities. The estimate of the collective dose to the population (as required by the European regulation) [1] is also included. Patients should be informed on the benefits of diagnostic and interventional procedures but also on the radiation risks. Referrers, radiologists and radiographers should be familiar with the dose quantities. The proper use of the radiation protection quantity “effective dose” (ED), with its uncertainties and limitations, is summarised in the review. ED allows the relative risk of different radiological procedures and different modalities to be compared and it is also used for the estimation of collective doses (population doses) derived from medical imaging as required by the European Directive on Basic Safety Standards [1]. The application of effective dose on the cumulative doses from recurrent imaging procedures in certain groups of patients, is considered as additional information to help in some aspects of justification and optimisation.

This review has been produced by the EuroSafe Imaging working group (WG) on “Dosimetry for imaging in clinical practice”, of the European Society of Radiology, to help understanding the patient dosimetry aspects in clinical imaging required by European and national regulations.

This is the content of the review:

1. Dosimetric quantities used in medical imaging and interventions for reporting patient doses and establishing diagnostic reference levels (DRLs).
2. Effective dose as a radiation protection quantity to compare different imaging modalities and to inform patients on the relative radiation risk.
3. Uncertainties and limitations on the use of effective dose in medical imaging.
4. Cumulative dose from recurrent imaging procedures and how this information may help to improve justification and optimisation.
5. Dosimetric information for practitioners, referrers and patients.

## Dosimetric quantities used in medical imaging for reporting patient doses (see Table 1)

**Table 1:** Quantities suitable for setting diagnostic reference levels (DRLs) ICRP-135 [4]

Image modality	Recommended quantity	Recommended unit
Radiography	Entrance-surface air kerma (K_a,e_) or Kerma-Area Product (P_KA_)	mGy or mGy.cm^2^
Mammography	Mean glandular dose (D_G_) or “average glandular dose” (AGD) and K_a,i_ (incident air kerma) or K_a,e_	mGy
Interventional fluoroscopy	kerma-Area Product (P_KA_) and air-kerma at the patient entrance reference point (K_a,r_)	Gy.cm^2^ and mGy
Computed tomography	CTDI_vol_ (computed tomography dose index) and Dose Length Product (DLP)	mGy and mGy cm
Nuclear medicine	Administered activity	MBq

The dosimetric quantity Kerma (or Dose) Area Product (P_KA_ or DAP) is used for radiography, fluoroscopy and interventional procedures and takes into account the radiation produced by the X-ray system and the irradiated area during the radiological procedure. It is usually measured in Gy.cm^2^. In most X-ray systems (especially for interventional radiology systems) a second dosimetric quantity, called air kerma (or dose) at the patient entrance reference point (measured in mGy) is also reported.

For projection radiography, in addition to P_KA_, the entrance surface air kerma (K_a,e_) (also known as entrance surface dose—ESD) is used, representing the radiation dose at the entrance of the patient, usually measured in mGy.

For Computed Tomography (CT), the Dose Length Product (DLP) is used. It takes into account the radiation produced by the scanner with the particular protocol of the examination and the scan range of the patient body examined. It is usually measured in mGy.cm.

$${\text{DLP}}\,\left( {{\text{mGy}}.{\text{cm}}} \right) = {\text{CTDI}}_{{{\text{vol}}}} \,\left( {{\text{mGy}}} \right) \times {\text{Scan}}\,{\text{Length}}\,\left( {{\text{cm}}} \right)$$

The Computed Tomography Dose Index (volume) (CTDI_vol_) (measured in mGy) is the parameter that best represents the average absorbed dose at a point within the scan volume for a particular scan protocol for a standardised phantom with 16 or 32 cm diameter [2].

The size-specific dose estimate (SSDE) can also be used for CT. SSDE considers corrections based on the size of the patient, using linear dimensions measured or determined from the patient or on patient images [3]. Dose estimates based on patient size are considered to be more accurate [4].

For mammography, the mean glandular dose is used, representing the mean absorbed dose in the glandular tissue of the breast, usually measured in mGy. Sometimes, the K_a,e_, measured in mGy, is also reported [4].

In nuclear medicine, the radioactive activity (usually measured in MBq) of the radiopharmaceutical administered to the patient is used.

It should be noted that the European Directive 2013/59/EURATOM on Basic Safety Standards [1] requires in the art. 58.b, that “Member States shall ensure that information relating to patient exposure forms part of the report of the medical radiological procedure”.

## Dosimetric quantities used in medical imaging for establishing diagnostic reference levels (DRLs)

A diagnostic reference level (DRL) is a form of investigation level used as a tool to aid in optimisation of protection in the medical exposure of patients for diagnostic and interventional procedures. It is used in medical imaging with ionising radiation, to indicate whether, in routine conditions, the amount of radiation used for a specified procedure (a clinical task) is unusually high or low for that procedure. For nuclear medicine, the administered activity (amount of radioactive material), or preferably the administered activity per unit of body weight, is used [4].

DRLs may be local, national or regional. The “local DRL” value is obtained from a few healthcare facilities, the “national DRLs” values are obtained from multiple facilities throughout a country and the “regional DRLs” values are obtained considering the national DRLs from a group of countries (a “region”).

Median values of distributions of DRL values of dosimetric quantities at a facility should be compared with DRL values. But values of DRL quantities should not be used for individual patients because the DRL process is intended for optimisation of protection for groups of patients and is based on standard and not individual patients. For individual patients, the dosimetric values may be higher or lower than the DRL. The priority in medical imaging should always be the appropriate image quality or diagnostic information for the involved clinical task.

Table 1 summarises the ICRP recommended dosimetry quantities and units used in medical imaging to establish DRLs [4].

## Effective dose (ED) as a radiation protection quantity

Effective dose was created by the ICRP to provide a dose quantity related to the probability of health detriment due to stochastic effects from exposure to low doses of ionizing radiation [5]. It is derived from the weighted sum of doses to tissues more sensitive to radiation and can only be derived by calculation. The tissue weighting factors are proposed by the ICRP and derived from epidemiological evidence. Effective dose is calculated for a “Reference Person” and not for an individual and is based on updated risk data and intended to apply as rounded values to a population of both sexes and all ages. Thus, for applications in medical imaging, the effective dose would be representative of a method and not individual (or even groups of) patients. Effective dose is not recommended for epidemiological evaluation [5, 6]. It should be noted that radiation detriment is only a part of the total health detriment in medicine.

Effective dose is defined as the sum of the absorbed dose by organs and tissues weighted by factors representing the specific radiosensitivities of each organ/tissue. This quantity can be related with the increases of cancer and hereditary effects for standard persons. The following tissue weighting factors (Table 2) have been proposed by the IRCP [5]:

**Table 2:** Tissues and weighting factors proposed by the ICRP to estimate effective doses [5]

Tissue	w_T_	∑ w_T_
Bone-marrow (red), Colon, Lung, Stomach, Breast, Remainder Tissues* (Nominal w_T_ applied to the average dose to 14 tissues)	0.12	0.72
Gonads	0.08	0.08
Bladder, Oesophagus, Liver, Thyroid	0.04	0.16
Bone surface, Brain, Salivary glands, Skin	0.01	0.04

^*^Remainder Tissues (14 in total): Adrenals, Extrathoracic (ET) region, Gall bladder, Heart, Kidneys, Lymphatic nodes, Muscle, Oral mucosa, Pancreas, Prostate, Small intestine, Spleen, Thymus, Uterus/cervix

The age distributions for workers and the general population (for which the effective dose is derived) can be quite different from that of the overall age distribution for the population undergoing medical procedures using ionizing radiation and will also differ from one medical procedure to another depending on the age-and-sex-prevalence of the individuals for the medical condition being evaluated [6].

## How to use effective dose to compare relative radiation risk from radiological procedures and imaging modalities

The ICRP states that effective dose can be of practical value for comparing the relative doses related to stochastic effects in the following cases [5]:

- Different diagnostic examinations and interventional procedures;
- The use of similar technologies and procedures in different hospitals and countries;
- The use of different technologies for the same medical examination, provided that the representative patients or patient populations for which the effective doses are derived are similar with regard to age and sex.

Table 3 shows some examples of effective doses from different imaging procedures from Germany [7] and the equivalent period of natural background radiation.

**Table 3:** Typical effective doses and equivalent of natural background radiation time for different imaging procedures used in Germany [7]

Imaging procedure	Typical effective dose (ED) in (mSv)	Approx. natural background radiation (for similar ED)
Chest PA	0.02	3 days
Abdomen AP/PA	0.34	2 months
CT-chest	5.1	2.4 years
CT-abdomen and pelvis	11	5.2 years
Coronary angiography	3.1	1.5 years
Percutaneous coronary intervention (PCI)	6.4	3 years
Endovascular aneurysm repair (EVAR) of the aorta	17	8 years
Lung perfusion scintigraphy 160 MBq Tc-99 m	1.8	10 months
Positron emission tomography (oncology) (350 MBq F-18-FDG)	4.6	2.2 years

The effective doses provided do not account for individual factors such as a patient’s sex, age or constitution. The dose uncertainty may exceed a factor of 5. Average natural radiation exposure in Germany: 2.1 mSv per year

Effective dose may be used to compare doses from different imaging modalities. But it should be noted that for the same technology (e.g., fluoroscopy guided procedures) the distribution of doses inside the patient may be very different depending on the quality of the X-rays beam (kV and filtration). For instance, the conversion factor to estimate effective dose from kerma area product (KAP) may increase in a 38% using high copper filtration in the X-ay beam [8].

The ICRP has published a new document on the use of dose quantities in radiological protection, with a chapter on the use in medical exposures [9]. Several scientific papers have been published in the last years dealing with this topic [10–15].

Table 4 summarises some relevant aspects to take into account when using “effective dose” in medical imaging.

**Table 4:** Summary of the relevant aspects to take into account for the use of effective dose in medical imaging

Relevant aspect	Interest for
Effective dose to “reference persons” can be used for comparing different imaging modalities	Comparing
Useful for classifying different types of imaging procedures for communicating risks to clinicians and to patients	Risk communication
Useful to inform decisions on justification of imaging procedures, planning research studies, and evaluation of unintended exposures	Justification
Uncertainties should be considered and variation in risk with age, sex and population group	Uncertainties
Situations in which a single organ receives the majority of the dose	Dose distribution
Organ-tissue doses, age and gender	Risk estimations

The new ICRP publication [9] suggests using the terminology that ED allows “approximate indicator of possible risk” with the additional consideration of variation in risk with age, sex and population group.

The amount of radiation and its distribution within the tissues of the body can be very different for several imaging modalities, even when a similar region of the body is being exposed. Since dose distributions from x-ray and nuclear medicine procedures are very different, the effective dose is suitable for use in straightforward comparisons of doses from different techniques [9].

Effective dose is not the best quantity for making comparisons between doses for similar techniques applied in different departments or institutions. Modality-specific dose quantities (e.g., P_KA_, DLP, CTDI_vol_) should be used for this purpose. However, in circumstances in which the dose distributions within the body may be substantially different between procedures, effective dose may provide an appropriate measure for comparison [9].

A chest CT examination and a conventional chest x-ray, both irradiate the lungs, but the effective dose from CT can be a few hundred times that of chest radiography, depending on the protocol technique (see Table 3). Referrers and practitioners should decide if such substantial difference in radiation dose is justified, for each individual patient.

The radiation risk of 1 mSv is not the same for a child of 10 years, or an adult of 25 years or an adult of 70 years. And it is not the same for a man or for a woman.

Lifetime risk of cancer incidence per Sv may be around twice as great for the 0–9 year’s age at exposure group than for the 30–39 years group. For patients in their 60 s, the lifetime risks from most imaging examinations are estimated to be about half those for patients in their 30 s, falling to less than one-third for patients in their 70 s and about one-tenth for those in their 80 s [9].

## Estimation of collective doses from medical imaging

The European Directive on Basic Safety Standards 2013/59/EURATOM [1] requires members of the European Union to make an estimation of population doses: “Member States shall ensure that the distribution of individual dose estimates from medical exposure for radiodiagnostic and interventional radiology purposes is determined, taking into consideration where appropriate the distribution by age and gender of the exposed”. Note that it is required the distribution by age and gender of the exposed population.

Collective effective dose is not intended as a tool for epidemiological risk assessment, and it is inappropriate to use it in risk projections. The aggregation of very low individual doses over extended time periods is inappropriate, and in particular, the calculation of the number of cancer deaths based on collective effective doses from trivial individual doses should be avoided [5].

**Fig. 1:**
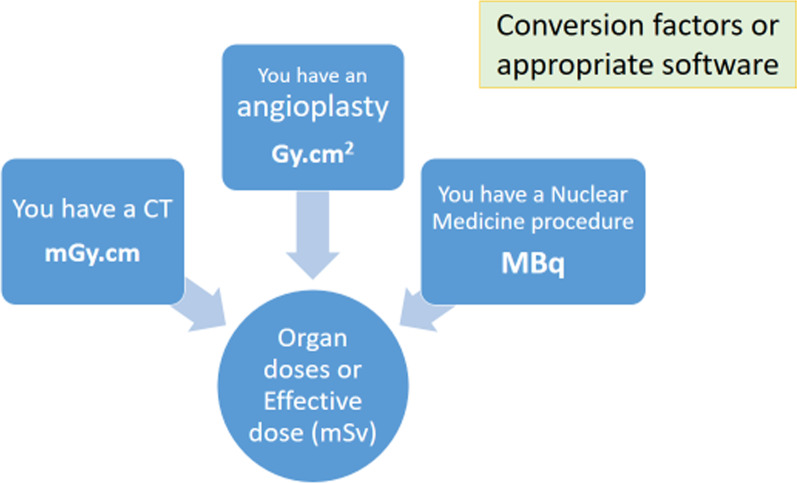
Estimation of effective doses from different imaging modalities using the conversion factors

UNSCEAR (United Nations Scientific Committee on the Effects of Atomic Radiation) periodically requires different countries, in a survey on medical exposures, to provide the number of different diagnostic and interventional procedures with the used radiation doses. In these surveys it is also possible to include the effective dose estimations for the main groups of procedures. UNSCEAR also has a “User Manual” for the “Global survey of radiation exposure” [16] including the conversion factors to estimate effective doses from the dosimetric quantity reported by the X-ray imaging systems. Figure 1 shows a diagram on how to estimate effective doses from different imaging modalities using the conversion factors.

## Uncertainties and limitations for the use of effective dose

The main uncertainties when trying to apply effective dose to individual patients are the intrinsic uncertainties derived from the Linear-Non-Threshold (LNT) model, when applied for diagnostic imaging, and for the risk quantification.

Uncertainties in both, effective dose calculation and radiation risk estimates, should be considered. The relative uncertainty in estimated values of ED for medical exposures for a reference patient is estimated to be about 40%. The estimated risk of cancer may be a factor of three higher or lower when applied to a reference patient and will be more variable when applied to an individual [13, 17].

For nuclear medicine examinations, the uncertainties in the relative values of ED for a reference patient are of the order of ± 50%. This is because of the tendency for doses to single organs (in particular the bladder) to be a substantial part of ED for some procedures. Prediction of risks of cancer for a reference patient derived using ED may be a factor of three higher or lower in practice [14].

The assessment and interpretation of effective dose from medical exposures of patients also needs to consider that some organs and tissues receive only partial exposures or a very heterogeneous exposure, which is the case especially with diagnostic and interventional procedures [5]. Stochastic risks estimations for individual organs may also be problematic for individual patients.

Stochastic risks are not modulated by dose rate, adaptive response, cellular repair of sub-lethal radiation damage, or genomic instability over long time periods (years) and ED applies only to an age-averaged, gender-averaged (male plus female) and region-averaged reference model [11].

In a recent paper on the “Past, present, and future of effective dose in Medicine”, Martin et al. highlighted that ED is based on reference phantoms representing average individuals, but this is often forgotten in its application to medical exposures. The dose received by patients will differ from that assessed for reference phantoms, and the risk per Sv depend on the age and gender.

Newer techniques and the proliferation of phantoms of increasing sophistication are allowing for the refinement of calculations of more “patient-specific or size specific ED” using reference anatomical phantoms. But such values do not conform to the ICRP definition of ED, based on the reference phantoms, and need to be distinguished from the reference quantity [15].

## Cumulative dose from recurrent imaging procedures in some groups of patients and its limitations in practical use

Several papers on recurrent imaging procedures with ionising radiation on the same patient have been published in the recent years [18–23]. Authors have focussed on patients undergoing recurrent CT exams that leads to cumulative effective dose (CED) of ≥ 100 mSv. Published data on the number of patients with cumulative effective doses ≥ 100 mSv ranged from 0.6 to 3.4% in CT and around 4% in interventional radiology. Eighty per cent of patients having a CED of ≥ 100 mSv had an oncological disease [18, 22].

The topic is relevant to improve justification and optimisation for the imaging procedures in the group of patients with high CED dose values. However, some authors [24] have alerted on several issues as if previous diagnostic radiation exposures should affect decisions on future examinations, concluding that bringing dose history into the decision process for justifying examinations may be not relevant for radiation risk and, rather than improving patient safety, would unnecessarily restrict access to radiation-based diagnostic examinations. In any way, the clinical context should be considered when highlighting the risks.

Getting 10 CTs during one hospital stay in a few days is different in radiation risk from getting 10 CTs to follow disease over a period of 10 years. Cumulative doses from recurrent exposures may be useful information but converting these cumulative doses into radiation risks for individual patients should be avoided. The IAEA is preparing a “Joint Position Statement and Call for Action for strengthening radiation protection of patients undergoing recurrent radiological imaging procedures”.

Dose management systems (DMS) may have a relevant role alerting referrers, radiologists and radiographers on previous examinations to profit from the existing diagnostic information and help to select the best imaging modality and protocol to be used in future [25]. Radiation doses may also be useful to inform patients on the benefits of the procedures and radiation risks, and on the potential need of a clinical follow up for interventional procedures if the skin doses may be near trigger levels [26, 27]. Refinements in the application of the justification criteria for these groups of patients should be considered.

One difficulty in many countries is the lack of a central data base for patient dose values and the difficulty to get the dose values from procedures carried out in different hospitals. Patient DMS may alert on cumulative high doses and this information should be available to the referrers and practitioners [28].

It should be noted that in the European Union, the directive 2013/59/EURATOM [1], requires individual optimisation, the evaluation of patient doses for some of the X-ray examinations and the capacity to transfer the information on the relevant parameters for assessing the patient dose, to the record of the examination. Information relating to patient exposure should be part of the report of the medical radiological procedures.

Sometimes referrers have little if any knowledge of patients’ imaging histories, individual CED can be useful. With this information, radiologists, referrers and radiographers can make rational decisions regarding further imaging safely. Of course, critically ill patients, will never face delays or denials of CT studies in life-threatening situations [29]. The decision for any imaging procedure should always be taken by the qualified practitioner and the estimated cumulative dose (if available) should never be an impediment to perform an imaging procedure if it is clinically indicated.

Cumulative dose may be used to refine, in some cases, the justification criteria (at the 3rd level, for individual patients) and the optimisation criteria for the next coming procedures to use low dose protocols if possible, and in some interventional procedures, using strategies to avoid skin radiation injuries. It is relevant to have alerts in the cumulative dose in the DMS but it is important to be aware that these systems are not always inter-connected between different hospitals in a city and country.

Personalised criteria for radiation protection in some patients (in parallel with the current approaches of the ‘personalised medicine’) should also be considered. The opinion of the patient needs to be taken into account. Some patients may accept additional radiation risks to confirm a diagnosis (e.g., a new CT). The result of this additional procedure may involve a relevant psychological benefit for the patient [30]. This may be considered as an ethical value concerning the patient autonomy to accept the radiation risk.

## Dosimetric information for practitioners, referrers, and patients

Effective dose can be used for patients when explaining possible radiation risks. Comparisons with other sources of exposure, such as background radiation or the dose from cosmic rays during air travel may be useful in some cases. But quoting values for the risk to patients is not recommended as a general approach, both because of the uncertainties and the fact that this creates the impression that the risk is known accurately [15]. It could be said that a dose of 10 mSv carries a nominal excess risk of < 1 in 1000, adding only slightly to the risk of developing cancer [15].

**For patients:** The suggested option for the Working Group on “Dosimetry for imaging in clinical practice” was to inform patients on “the dose values and units, reported by the X-ray system”. Other potential options, as informing on the effective doses derived from the imaging procedures or the estimation of the equivalent time in background radiation, were not considered appropriate at this step [27].

A brief explanation on the used dosimetric quantities and units included in the examination imaging report should be available for patients (e.g., what is a “dose area product” or a “dose length product” or the activity of a radionuclide).

If several imaging modalities may be used for a patient, effective dose may be used for comparison purposes but insisting that this quantity cannot be used to estimate individual radiation risk.

The proper information on radiation risk for patients (and its uncertainties) may be a critical issue to avoid unjustified patient fear. The individual health aspects should be part of this information considering the acceptability of the risk by the patient waiting for a good diagnosis [27].

**For practitioners**: More detailed information on dose values from the previous examinations may be useful to confirm the justification of the requested procedure, to confirm that the images and the reports are available and to decide the appropriate protocol (optimisation) for the new examination. This might be useful to consider non ionising radiation imaging modalities, namely MRI and Ultrasound in replacement of Ionising radiations, and this should be discussed with the referrers. For interventional procedures, the trigger levels need to be considered in the case of potential clinical follow-up. If new interventional procedures may be necessary, the details of the previous ones could help in avoiding potential skin radiation injuries.

**For referrers**: To also know the estimation of effective doses, or in some cases organ doses (e.g. mammography), may be of value to indicate which imaging modality to use depending on the diagnostic information to be obtained and previous examinations that have already taken place. Referrers should be aware of the benefits of MRI and Ultrasound, especially when they could replace Ionising radiation techniques. Estimations of effective doses (and the age and gender of the patients) may be useful to communicate the radiation risks and the benefits of new examinations. The uncertainties in all the dose estimations need to be considered.

## Data Availability

All information contained in this review is from scientific papers, reports from international organisations (all referred in the references section) and opinions of the authors.

## Abbreviations

AGD: Average glandular dose
AP: Anteroposterior
CED: Cumulative effective dose
CT: Computed Tomography
CTDIvol: Computed Tomography Dose Index (volume)
DAP: Dose Area Product
D_G_: Glandular dose
DLP: Dose Length Product
DMS: Dose management systems
DRL: Diagnostic reference level
ED: Effective dose
ESD: Entrance surface dose
ET: Extrathoracic
EVAR: Endovascular aneurysm repair
Gy: Gray
ICRP: International Commission on Radiological Protection
Ka,e: Entrance surface air kerma
Ka,i: Incident air kerma
Ka,r: Air-kerma at the patient entrance reference point
KAP: Kerma area product
LNT: Linear-Non-Threshold
MBq: Megabecquerel
mGy: Milligray
PA: Posteroanterior
PCI: Percutaneous coronary intervention
P_KA_: Kerma area product
SSDE: Size-specific dose estimate
Sv: Sievert
WG: Working group
